# “We Are Not Really Marketing Mental Health”: Mental Health Advocacy in Zimbabwe

**DOI:** 10.1371/journal.pone.0161860

**Published:** 2016-09-08

**Authors:** Reuben Hendler, Khameer Kidia, Debra Machando, Megan Crooks, Walter Mangezi, Melanie Abas, Craig Katz, Graham Thornicroft, Maya Semrau, Helen Jack

**Affiliations:** 1 Arnhold Institute for Global Health, Icahn School of Medicine at Mount Sinai, New York, New York, United States; 2 Kushinga, Harare, Zimbabwe; 3 Department of Psychiatry, University of Zimbabwe, Harare, Zimbabwe; 4 Department of Psychology, Women’s University in Africa, Harare, Zimbabwe; 5 Institute of Psychiatry, Psychology and Neuroscience, King’s College London, London, United Kingdom; 6 Harvard Medical School, Boston, Massachusetts, United States; Stellenbosch University, SOUTH AFRICA

## Abstract

**Introduction:**

Few people with mental disorders in low and middle-income countries (LMICs) receive treatment, in part because mental disorders are highly stigmatized and do not enjoy priority and resources commensurate with their burden on society. Advocacy has been proposed as a means of building political will and community support for mental health and reducing stigma, but few studies have explored the practice and promise of advocacy in LMICs.

**Methods:**

We conducted 30 semi-structured interviews with leaders in health and mental health in Zimbabwe to explore key stakeholder perceptions on the challenges and opportunities of the country’s mental health system. We coded the transcripts using the constant comparative method, informed by principles of grounded theory. Few interview questions directly concerned advocacy, yet in our analysis, advocacy emerged as a prominent, cross-cutting theme across participants and interview questions.

**Results:**

Two thirds of the respondents discussed advocacy, often in depth, returning to the concept throughout the interview and emphasizing their belief in advocacy’s importance. Participants described six distinct components of advocacy: the advocates, to whom they advocate (“targets”), what they advocate for (“asks”), how advocates reach their targets (“access”), how they make their asks (“arguments”), and the results of their advocacy (“outcomes”).

**Discussion:**

Despite their perception that mental health is widely misunderstood and under-appreciated in Zimbabwe, respondents expressed optimism that strategically speaking out can reduce stigma and increase access to care. Key issues included navigating hierarchies, empowering service users to advocate, and integrating mental health with other health initiatives. Understanding stakeholder perceptions sets the stage for targeted development of mental health advocacy in Zimbabwe and other LMICs.

## Introduction

Mental disorders account for 7.4% of the global burden of disease, measured in disability-adjusted life years (DALYs) [1], yet mental health receives a median 2.5% of health-related expenditures worldwide and only 1% in low-income countries. 70% of African countries spend less than 1% [2]. As a result, the vast majority of people with mental disorders in low and middle-income countries (LMICs) go untreated [3–5], even though cost-effective psychosocial and pharmacological treatments have been developed and tested in LMICs [6–8]. This mental health treatment gap reflects a global lack of commitment to providing adequate financing and support for the prevention and treatment of mental disorders.

Allocating resources between competing priorities is often a deeply political process, and the importance of political will for global health has long been recognized [9–12]. The development of political will for mental health is stymied by stigma, scarce resources, controversy over the nature of mental disorders, and limited visibility of the disease burden [13–16].

Advocacy is widely recognized as a tool for generating local and global support for health issues. According to the World Health Organization (WHO), the concept of mental health advocacy subsumes “various actions aimed at changing the major structural and attitudinal barriers to achieving positive mental health outcomes in populations” [17]. One important thread in mental health advocacy responds to the stigma people with mental disorders face by promoting human rights and respecting service users’ autonomy as advocates for themselves and each other [18, 19]. Advocacy has proven successful in the context of other major global health challenges, such as HIV/AIDS. For instance, in 1998, a small group of activists in South Africa sued the government for refusing to scale up a program for the prevention of mother to child transmission. Despite stigma associated with the HIV/AIDS epidemic, the activists won the case, forcing the government to make life-saving antiretroviral therapy available to pregnant mothers and neonates and helping usher in universal access to treatment [20].

The WHO considers advocacy a pillar of its comprehensive strategy for strengthening mental health systems, aligned with other central topics such as financing and organization of services [17]. The WHO’s report, *Advocacy for Mental Health*, outlines in practical terms how service users, families, NGOs, healthcare providers, and policy-makers can all participate in and support advocacy. Elsewhere too, researchers and practitioners have proposed advocacy as a potential method of generating political will, adequate resources, and community support for mental health service provision [21]. In partnership with service users and advocates, several NGOs have constructed toolkits for mental health advocacy [22, 23]. These toolkits elaborate concrete procedures and strategies advocates can use to disseminate messages and influence policy.

Despite these high-profile calls for advocacy, its effectiveness for generating political will and community support for mental health in LMICs remains largely untested. The WHO report lists but a few small-scale examples of successful advocacy efforts in LMICs [17]. In high-income countries (HIC), service user and family driven advocacy has successfully educated the public about mental health, generated funding for research into mental health treatments, and spurred legislation outlawing discrimination and protecting rights [24–26]. However, the context for advocacy differs between HICs and LMICs in terms of resources available for health, political contexts, and cultural understandings of mental health.

Not only is advocacy’s effectiveness largely untested—few studies have explored what health advocacy looks like [27, 28], and even fewer have focused specifically on advocacy for global mental health [29–32]. One qualitative study explored stakeholder perceptions of why mental health is a low priority and what to do about it in Ghana, South Africa, Uganda, and Zambia [33]. Another study, based on interviews with eleven stakeholders in seven African countries, showed that the process itself of service users organizing together as advocates may help restore to them a sense of community and belonging [31]. A third qualitative study documented how a mental health advocacy coalition in Sierra Leone built relationships among stakeholders and with decision-makers to improve national policies, scarce resources notwithstanding [32].

Despite the limited evidence on advocacy efforts, interventions are being designed and implemented to train mental health leaders in advocacy. The *Mental Health Leadership and Advocacy Program* in Ibadan, Nigeria, which combines a centralized training program for leaders in mental health with capacity building efforts for country-specific stakeholder groups, has reached over 100 participants and engaged stakeholder groups in West Africa since its inception in 2010. The advocacy course resulted in the development or strengthening of a stakeholders’ advisory council in each participating country, which calls for greater support for mental health and brings additional perspectives to policy and implementation decisions [29].

This paper aims to examine how key stakeholders in Zimbabwe think about mental health advocacy. It is appropriate to examine advocacy in Zimbabwe because Zimbabwe exemplifies a problem that many LMIC countries share: inadequate mental health resources. Moreover, Zimbabwe’s longstanding mental health policy and recent efforts to build mental health research and teaching capacity would make officials and clinicians more likely to have thought deeply about mental health at a systems level than in other settings. The results of this study can inform the development of interventions aimed at supporting mental health advocacy in Zimbabwe and other LMICs.

## Methods

### Setting

Zimbabwe’s population exceeds 14 million, and researchers estimate the prevalence of mental disorders at more than 15% [34, 35]. However, Zimbabwe has only eleven psychiatrists, most of whom practice in Harare, the nation’s capital. The country has two psychiatric hospitals, which provide long-term, institutionalized care: Ingutsheni, in Bulawayo, the second largest city, and Ngomahuru, in the rural area of Masvingo. Like many countries in sub-Saharan Africa, Zimbabwe is moving away from institutionalized mental health care and toward providing mental health services in community and general hospital settings. Mental health services at public facilities and in the community are funded by the government and provided free of charge to patients, yet workforce constraints, variable medication availability, and limited community infrastructure restrict service provision. Zimbabwe also has robust private sector mental health services accessible to a small segment of the population, and many mental health professionals work at least part-time in private practice.

The University of Zimbabwe offers post-graduate medical training programs in psychiatry that have been recently expanded [36, 37]. Only one hospital, Ingutsheni, trains psychiatric nurses. In addition to or instead of seeking mental health care from conventional healthcare providers, many people in Zimbabwe consult traditional or faith healers. In this study, we drew our sample from these diverse settings in which mental health care is provided in Zimbabwe.

### Study design and sample

We conducted semi-structured, qualitative interviews with practitioners and policymakers who work at the national level in mental health or related healthcare fields in Zimbabwe about their perceptions of the country’s mental health system (n = 30; see Table 1). Qualitative interviews were appropriate because this study was an initial exploration of the topic and we wanted to explore stakeholder perspectives in depth and with nuance.

**Table 1:** Characteristics of interview respondents.

Profession	Number of participants	Institution	Number of participants
Physician	3	NGO	5
Psychiatrist	4	General hospital	5
Researcher	2	Psychiatric hospital	3
NGO worker	4	Government	8
Administrator	2	University	6
Nurse	5	Prison Service	2
Policymaker	8	Traditional healing facility	1
Traditional healer	1		
Psychologist	1		
**Total**	30	**Total**	30

To recruit interview respondents, we used the chain referral or ‘snowball’ method [38]. This recruitment method was appropriate because the mental health community within Zimbabwe is small and interconnected, yet not clearly defined in any source or well known outside the community, and because we were seeking an exhaustive sample of national-level stakeholders. To recruit participants, we first spoke with the Deputy Director of Mental Health Services at the Ministry of Health and Child Care and with the Chairman of the Department of Psychiatry at the University of Zimbabwe and asked them to recommend people who are key stakeholders in mental health policy and systems design. We then asked every subsequent interview respondent to recommend other people we should interview and contacted those people. After conducting approximately half of the interviews, we held a stakeholders’ meeting, during which we brought together mental health researchers, policymakers, and practitioners to discuss the preliminary findings of the study and possible next steps. We asked the group at the stakeholders’ meeting to suggest additional interview respondents, whom we subsequently contacted. Only one interview conducted after the stakeholders’ meeting involved someone who had been present at the meeting; with that one exception, it is unlikely the stakeholders’ meeting influenced interview content. Based on the suggestions of our interview respondents and our research team’s knowledge of Zimbabwe’s mental health system, we believe that we interviewed a nearly exhaustive sample of national leaders who were involved in mental health services and worked within traditional power structures and hierarchies.

Service users are often excluded from these power structures, even though service user voices are especially important to discussions of mental health [18, 39]. We did not solicit the participation of service users for two reasons. First, we aimed to better understand the mental health systems and policies and how different sectors within mental health services connected, which we thought would best be accomplished by speaking with leaders of those institutions. We believed that this understanding was a prerequisite to conducting a study that focuses solely on the voices of service users and includes a variety of perspectives from an often vulnerable and marginalized community. Second, we were concerned that service users, a vulnerable population, might face stigma or reprisal should their participation in the study be discovered.

Before starting the interview, we explained the study to each participant and obtained written consent. This study received ethical review and approval from the Medical Research Council of Zimbabwe (MRCZ/B/512), the Joint Research Ethics Committee (JREC 161/13), the Departmental Research Ethics Committee of the Oxford Department of Politics and International Relations (DPIR/C1A/12-054), and the Icahn School of Medicine at Mount Sinai (14–00066). All authors who participated in data collection or analysis were covered by one or more of these institutional reviews.

### Data collection

We adapted an interview guide, with permission, from the Emerald programme [40], a multi-country mental health systems strengthening initiative. The interview guide asked respondents about the greatest challenges and opportunities within mental health in Zimbabwe across the following topics: mental health law and policy, coordination and consultation, integrated mental health services, human resources capacity, financing, monitoring and evaluation, and quality assurance and ethics. Within each topic, the interviewer asked open-ended questions and then more specific follow-up questions about respondents’ statements and in reference to common elements of mental health systems. The theme of advocacy was addressed in a very narrow context within the interview guide (i.e. one question for practitioners about advocating on behalf of patients, and one about whether service users can voice grievances) and primarily emerged from participants’ responses to other questions.

Three researchers (KK, HJ, and DM) with training and experience in qualitative interview technique [41] conducted and digitally recorded the interviews. Interviews lasted 30–90 minutes, and most were conducted at a participant’s work place or a public café. Twenty-eight interviews were conducted in English. Two interviews were conducted in a combination of English and the indigenous language Shona by an interviewer fluent in both languages (DM). All interviews were professionally transcribed. Interviews conducted in a combination of Shona and English were transcribed verbatim and then translated into English.

### Data analysis

Data analysis was conducted using the constant comparative method, adapted for health services research [42, 43] and informed by principles of grounded theory [44].

Two researchers (HJ, KK) separately reviewed four transcripts and inductively assigned a label, or code, to every new theme that emerged from the data. They then compared their code assignments to develop a code list that captured all of the themes present in the data. Two researchers (two of DM, MC, and RH) separately used this code list to assign codes to the remaining transcripts. During the coding process, two additional codes were added to reflect new themes that emerged from the data. Coders returned to the initial transcripts to review them for presence of the themes from the added codes. After assigning codes, the two researchers reconciled their code assignments through discussion. One researcher (HJ or KK) reviewed all reconciled transcripts to help ensure consistency of code assignments across transcripts. Coded transcripts were entered into NVivo (version 10 for Windows) for data retrieval, comparison of codes, and model generation.

Advocacy emerged definitively as a cross-cutting theme in responses from diverse respondents and across interview topics. Participants discussed advocacy both as a main idea and in reference to varied additional themes. For this paper, we limited our analysis to parts of the interview that were coded as “advocacy.” Quotes were coded as “advocacy” if they described speaking up about mental health, the state of awareness about mental health and efforts to raise awareness, or going to people in power to make requests about mental health needs. Two researchers (HJ, RH) created a set of subcodes to explore the themes within the “advocacy” code and inform this analysis.

For the ideas that emerged most strongly from coding, we computed “signal strength”–the number of participants who endorsed each idea. We report signal strength to clarify the degree to which ideas were widespread within our sample.

## Results

Two thirds of the stakeholders we interviewed (n = 20) discussed advocacy, often in depth, returning to the concept again and again, conveying a breadth of interesting ideas, and emphasizing their belief in advocacy’s importance. Participants talked about six distinct components of advocacy (Table 2). Prominent ideas within each of these themes are displayed in Table 3, alongside illustrative quotes and a computation of “signal strength”—the number of participants endorsing each idea. Where relevant, particularly salient illustrating quotations are also provided in text below.

**Table 2:** Components of advocacy that respondents discussed.

**Advocates:** The people or groups who advocate
**Targets:** Whom the advocates approach with their asks
**Asks:** What the advocates want to achieve
**Access:** How the advocates reach their targets
**Arguments:** How the advocates make their asks
**Outcomes:** The results of advocacy

**Table 3:** Themes, illustrative quotes, and signal strength.

Concept	Illustrative quotes	Signal strength^1^ (out of 30 respondents)	Number of health professionals endorsing the statement^2^ (out of 15 health professional respondents)
**Advocates**			
Service users	“First is to empower the consumer, they have to know their rights; they have to know what the government is supposed to be providing. In fact, they have to hold the government accountable for their service.”–NGO worker	10	5
Health professionals	“In most cases things happen because service providers are actually advocates for clients.”–Nurse	7	6
	“We are still concentrating on providing clinical services in institutions and we are not going beyond that.”–Psychiatrist		
Government	“I went for a supervisory visit in one of the hospitals and I found a patient who had tried to commit suicide and [was] locked in a secluded…ward and [was] not being attended to. Then I called the head of the hospital and I raised the issue.”–Policymaker	6	1
NGOs	“It’s the primary mandate of the government to look after them and us as an organization we can’t like take over so we lobby we advocate for everything.”–NGO worker	5	0
**Targets**			
Community	“I also think the part of the problem is we are not really marketing mental health.”–Psychiatrist	22	12
Government	“The government has a number of competing you know… priorities so unless the mental health sector makes adequate noise, demand[s] the services, they will continue to address HIV and all because the people are always in the streets everyday to say we need ARVs.”–NGO worker	18	10
Donors	“We need partners because at the moment, there are very few partners that are assisting.”–Nurse	12	7
Health professionals	“I think a large percentage of doctors don’t appreciate that we’ve [psychologists] got a role to play, until you solve a problem they see, and once you’ve done that you begin to, you develop working you know relations with and they start you know, referring.”–Psychologist	11	8
**Asks**			
Community awareness	“I think the major barrier of the major problem is that people are ignorant about what psych is and how important it is. But the moment you explain to people, people appreciate, even if they are not trained in psychiatric. They appreciate the importance of it.”–Physician	20	11
	“We are trying to capitalize on educating, empowering the patients before they go home. Empowering the family of the patient before they go home so that at least they know how the community would support the people with mental illness.”–NGO Worker		
More resources or priority for mental health	“I also meet the permanent secretary talking about the shortages of drugs. Then I always get responses although I know that it is very difficult for the drugs to be purchased because there is no money.”–Policymaker	17	7
Protection of the rights of individual service users	“We had to write a letter to the courts to say she is recovering so only she be allowed to marry and so she has since married.”–Psychiatrist	9	7
**Access**			
Use established processes or hierarchies to reach targets	“You give them the respect which is due to them you follow the, whatever standard they need you to follow. I think as people on the ground that’s our strength: we are never in conflict with them.”–NGO worker	13	7
Do direct outreach to targets	“I’m very open and I explain the ministry of health the ministry of justice we need rehabilitation center.”–Psychiatrist	9	5
Hold public events	“We have platforms like Agricultural Show where people go and display what they are doing. I think we, whenever there is such an opportunity where you can go out and try and you know report yourself to the public.”–Psychologist	8	6
Use the media to reach the community	**“**I think it would be something that involves setting up a media campaign with a very specific objective and we don’t necessarily have to target every single condition. We can target the most common condition like depression and really come up with a marketing strategy on how to make people more aware of depression.”–Psychiatrist	3	2
**Arguments**			
Relate mental health to other problems people face	**“**When you talk about mental illness people think that you are talking about people who are lunatic but mental illness it covers a lot of things that affects people on day to day basis that may not be thought by people as part of the mental illness or part of mental health.”–Physician	7	4
Use data	“If there is no evidence gathered, people will just continue to say no.”–Policymaker	7	4
	“First you take a position and you do a survey come up with a documentation, approach the ministry, you see ‘we have come up…’”–Physician		
Raise awareness and assume support will follow	“If community can understand you know, maybe something changes.”–Psychiatrist	5	3
	“Knowledge is very key in terms of driving people.”–Policymaker		
**Outcomes**			
Advocacy often fails	“WHO if the government has not indicated that there is need, no matter how much you would cry for that medication, they will not act.”–NGO worker	11	4
There are examples of when advocacy has succeeded	“They wanted to turn a mental health unit into a TB unit…we actually had a patient who called whose son was being refused admission…we directed them to the permanent secretary to go and register their concern but we during that process…expressed our concern in the media…so out of that the decision was therefore reversed.”–NGO worker	9	5
Hierarchical decision-making is a barrier to success	“By right we put our request through, through our most and next senior like I answer to the Principal Director and my Principal Director is very supportive. And he is always approving whatsoever I request. And then from there it goes to the administration side that’s where we don’t get positive responses.”–Policymaker	3	1

^1^“Signal strength” is the number of participants who endorsed a given statement or idea. This is not a representative sample, but the magnitude of support for a theme provides some insight into how widespread an idea is within the sample.

^2^The number of health professionals amongst respondents who endorsed a given theme or statement. We labeled as a health professional each respondent with significant experience in direct patient care, whether as a psychiatrist, physician, nurse, psychologist, or traditional healer. We separated out this group to determine how ideas might differ between those with and without direct patient care experience.

### Advocates

Respondents identified five groups who advocate for mental health: service users, their families, healthcare providers, non-governmental organizations (NGOs), and government officials. Of these, respondents primarily discussed advocacy by service users and by healthcare providers.

Advocacy by service users is uniquely important, according to many respondents. Service users are uniquely positioned to set advocacy priorities because of their firsthand knowledge of mental health systems. Their affecting personal stories can be especially influential. Some said the process of participating in advocacy can itself be therapeutic for service users. Many respondents lamented uneven implementation of systems for gathering service users’ input, the underdeveloped state of patient associations, and how ignorance of their rights prevents service users from supporting and defending themselves and each other.

“First is to empower the consumer, they have to know their rights; they have to know what the government is supposed to be providing. In fact, they have to hold the government accountable for their service.”

–NGO worker

Several respondents expressed optimism that service users could become more involved in advocacy despite barriers like being institutionalized, the belief they would not be listened to, and mental illness itself. Two nurses, however, also voiced concern that service users might abuse knowledge of their rights to utilize health resources inappropriately or act out against healthcare providers.

The majority of healthcare workers we interviewed described important roles that nurses and psychiatrists play in advocating for individual service users and system-level changes.

“In most cases things happen because service providers are actually advocates for clients.”

–Nurse

A number of psychiatrists recognized that they need and want to advocate more for system-level change, but have little capacity because of their clinical, teaching, and research responsibilities. Providers also cited lack of confidence in their work and doubts about the potential of advocacy as barriers to advocating for broader changes. A number of respondents thought that people who work in other disease areas, such as HIV and TB, had done a much better job raising public awareness and wanted mental health to emulate these models and reach the same status.

### Targets

Respondents highlighted four groups to whom they advocate: the government, international donors, health professionals, and the community.

Almost uniformly, participants discussed requesting support from the government. They primarily approached government officials with specific requests about basic services, such as operational expenses at a hospital or increased human resources at a clinic. Most participants understood the government to be segmented. The officials in the government who were in charge of mental health services were not the target of most advocacy, as they had limited control over resources. Instead, many respondents described approaching officials who managed the budget or oversaw human resources, either at the national or local level, and many indicated that these individuals had little knowledge of or interest in mental health.

“The government has a number of competing you know… priorities so unless the mental health sector makes adequate noise, demand[s] the services, they will continue to address HIV and all because the people are always in the streets everyday to say we need ARVs.”

–NGO worker

While most respondents saw the government as the primary provider of resources, some in leadership roles also discussed approaching donors, primarily international organizations, whom many called “international partners.” Whereas stakeholders mostly described approaching the government for general budget requests, those who approached donors discussed soliciting support for specific projects, such as revising the Mental Health Act or holding an awareness-raising event.

A number of respondents, particularly specialist mental health providers, spoke about raising awareness about mental health treatments within the healthcare professions. In general, respondents indicated that general health professionals need to be reminded of the importance of mental health, told to assess and treat mental health conditions in their patients, and taught to do so appropriately.

“I think a large percentage of doctors don’t appreciate that we’ve [psychologists] got a role to play, until you solve a problem they see, and once you’ve done that you begin to, you develop working you know relations with and they start you know, referring.”

–Psychologist

Nearly all respondents talked about the stigma or lack of awareness of mental health in the general population. More than half suggested that advocacy efforts to the community, in the sense of outreach and education, are necessary for those in need of mental health services to present for care and for service users to be supported outside hospital settings.

“Suppose we have a perfect ah, environment where we have got our institutions, the rehabilitations, the half way places, the staff that is required and drugs. You would still want to go a step further and then ensure that these people are able to stand up in society and say you know I can actually assume a normal life.”

–Policymaker

### Asks

Respondents emphasized that the goals of advocacy are to 1) raise awareness in the community, 2) increase the priority of mental health for the government, donors, and healthcare workers as reflected in more resources, and 3) improve treatment of service users clinically and by laypeople in the community. Participants described how, in some cases, advocacy takes the form of lobbying and persuasion, where advocates ask directly for what they need. In other cases, advocacy to government officials and donors takes the form of education or awareness-raising aimed at correcting assumptions about mental health, reflecting the belief that lack of prioritization results more from ignorance of the magnitude of mental health needs than from knowing disregard of those needs.

“I think the major barrier of the major problem is that people are ignorant about what psych is and how important it is. But the moment you explain to people, people appreciate, even if they are not trained in psychiatric. They appreciate the importance of it.”

–Physician

According to some participants, a central goal of advocacy is community education, aiming to inform about the nature and scope of mental health, convince people that mental health is important, reduce stigma, promote effective and humane caretaking, and facilitate appropriate use of medical treatment. One physician wished the community would conceptualize mental health more broadly than psychosis and take stock of its prevalence and impact in their lives.

“To me that is the biggest challenge, we need to change our orientation towards health. We need to embrace the Worlds Health Organisation definition of health. That is not just physical but there is mental and emotional component and the spiritual component.”

–Physician

Providers, NGOs, and government officials discussed advocating to the government and international donors for access to medications, appropriately trained providers, rehabilitation centers, improved living conditions in psychiatric hospitals, and support for community-based psychiatric treatment. Several respondents celebrated that diverse stakeholders had been consulted in drafting Zimbabwe’s Mental Health Act twenty years ago but noted a perceived need for revision. However, even more respondents felt advocating for better *implementation* was the highest priority related to the Act.

NGOs, families, and providers also championed the needs of individual service users. Providers advocated within the healthcare and legal systems for consideration of mental illness in criminal trials, for prompt review of involuntarily commitment of service users to psychiatric institutions, to help service users obtain access to treatment facilities, or to insist that service users’ rights be respected. One physician explained, for instance, that he had asked a court to allow a patient of his to get married.

“We had to write a letter to the courts to say she is recovering so only she be allowed to marry and so she has since married.”

–Psychiatrist

### Access

Respondents discussed a range of ways of gaining access to the targets of their advocacy efforts including via established processes or hierarchies and through media and other public outlets.

Most participants, particularly those in administrative or nursing roles, discussed approaching the government and health professionals only through established processes or hierarchies, such as submitting a written request for funding or relaying needs to a direct superior and having that person relay the request through subsequent superiors. They emphasized the importance of following the processes, and their comments often indicated that they did not think it was an option to work outside of them.

“You give them the respect which is due to them you follow the, whatever standard they need you to follow. I think as people on the ground that’s our strength: we are never in conflict with them.”

–NGO worker

Some respondents, however, said they are able to go more directly to officials in power, either people in government or donors. Those who are able to directly reach their targets are generally higher status or work in government themselves and access their targets because of existing relationships or because they are high enough status to call a meeting with the key players. However, service users also have the right to communicate directly with powerful officials:

“The mental health act actually says, the mental ill patient in his or her capacity can write straight to the president…people have to be educated and told that you have got this law and this is what it entails.”

–Nurse

Respondents working in hospitals and in government indicated that service users, many health professionals, and administrators use official channels, such as a process for voicing grievances with hospital care described in the Mental Health Act, a complaint box at the hospital, or an appeals procedure, to bring concerns to government. Some respondents, including many hospital officials, were worried that service users and their providers do not know they are able to make complaints in this way or that the law has not been adequately implemented so there are no mechanisms for officials to respond to service user grievances or appeals for release from a psychiatric facility. Many were concerned that the Mental Health Review Tribunal, the body that decides whether or not patients can be kept at the hospital against their will, meets very infrequently.

When approaching the community, respondents, particularly psychiatrists, people working in government, and NGO leaders, described using existing platforms, such as the media, public events like agricultural fairs, religious groups, and schools, to speak about the importance of mental health.

**“**I think [marketing mental health] would be something that involves setting up a media campaign with a very specific objective and we don’t necessarily have to target every single condition. We can target the most common condition like depression and really come up with a marketing strategy on how to make people more aware of depression.”

- Psychiatrist

### Argument

Stakeholders discussed a number of different ways of framing their requests. One of the more common strategies that participants proposed was relating mental health to concerns that the target already has. For instance, a number of psychiatrists thought it would be a good idea to explain to the public that some of the stories they see in the news, such as a man killing his wife, may have mental illness as a root cause.

“Every other day there is something related to mental health [in the] Daily News headlines. You know, woman jumps from the 13th floor of the building… but we are not doing what we should be doing as mental health practitioners to actually promote mental health in view of all those things that are happening.”

–Psychiatrist

Others thought it would be helpful to relate the country’s economic woes or political instability to the lack of attention to mental health when approaching the public or policymakers. Similarly, respondents, particularly those in government, talked about packaging information in a way that appeals specifically to certain groups, playing to their existing interests, concerns, and level of knowledge. When advocating to people within government or to donors, participants, particularly who worked in the government, discussed framing their request in terms of keeping Zimbabwe in compliance with WHO guidance or international conventions.

Many participants, especially psychiatrists and those working for NGOs, indicated that, particularly when approaching the government or donors, it is important to have data on disease burden, cost-effectiveness of treatment, the poor condition of hospitals or prisons, or attitudes about mental health. They felt unable to act effectively without this data, and often proposed data collection as a first step for advocacy.

“If there is no evidence gathered, people will just continue to say no.”

–Policymaker

Others indicated that involving service users in advocating for resources or community awareness helps humanize mental health and bring more attention to it.

“At times you really need someone, a consumer who is empowered to say ah it’s affecting me in this way and that way.”

–NGO worker

### Outcomes of Advocacy

Respondents relayed a number of anecdotes of successful advocacy. For instance, a national advocacy group petitioned the government during a recent drug shortage, and the government made more drugs available. Another example involved protecting existing mental health resources:

“They wanted to turn a mental health unit into a TB unit…we actually had a patient who called whose son was being refused admission…we directed them to the permanent secretary to go and register their concern but we during that process…expressed our concern in the media…so out of that the decision was therefore reversed.”

–NGO worker

However, respondents also described the frequency with which advocacy fails, often in categorical terms rather than with specific stories. Respondents indicated that the government often does not acquiesce to advocates’ requests for resources due to a lack of funding, capacity, or awareness about the need, or because of Zimbabwe’s complex political climate. Two respondents mentioned instances in which international donors did not respond to advocacy because they were not persuaded that mental health was a priority.

“WHO if the government has not indicated that there is need, no matter how much you would cry for that medication, they will not act.”

–NGO worker

Respondents cited defensiveness as an obstacle to advocacy success, because, in many cases, those mandated to address concerns are the very people implicitly critiqued in requests for change.

A number of participants, both health professionals and non-health workers, indicated that decision-making hierarchies get in the way of advocacy. At every level in a government, donor, or hospital hierarchy, an opportunity exists for someone to quash the request. For instance, nurses may not be able to redress concerns without physicians’ approval. Requests to the government often need to rise up through a series of officials before they are presented to decision-makers with the requisite authority over resources.

“By right we put our request through, through our most and next senior like I answer to the Principal Director and my Principal Director is very supportive. And he is always approving whatsoever I request. And then from there it goes to the administration side that’s where we don’t get positive responses.”

–Policymaker

Another theme that emerged was how different advocacy efforts impact one another. For instance, providers’ efforts to obtain resources from international donors are influenced by providers’ and service users’ advocacy for government to prioritize mental health, because international donors may align their giving with government priorities.

## Discussion

These findings provide an overview of key stakeholder perceptions of the role of advocacy in Zimbabwe’s mental health system and add to the growing international dialogue about how best to close the mental health treatment gap [17, 18, 21, 33, 45]. Even though few interview questions directly focused on advocacy, advocacy emerged through systematic analysis as a salient theme, suggesting that respondents consider advocacy an important way to promote mental health. Despite their perception that mental health is widely misunderstood and under-appreciated in Zimbabwe, respondents expressed optimism that speaking out strategically can change minds and improve healthcare. People can and do advocate for mental health despite limited resources, respondents said, but more advocacy is needed.

**Fig 1:**
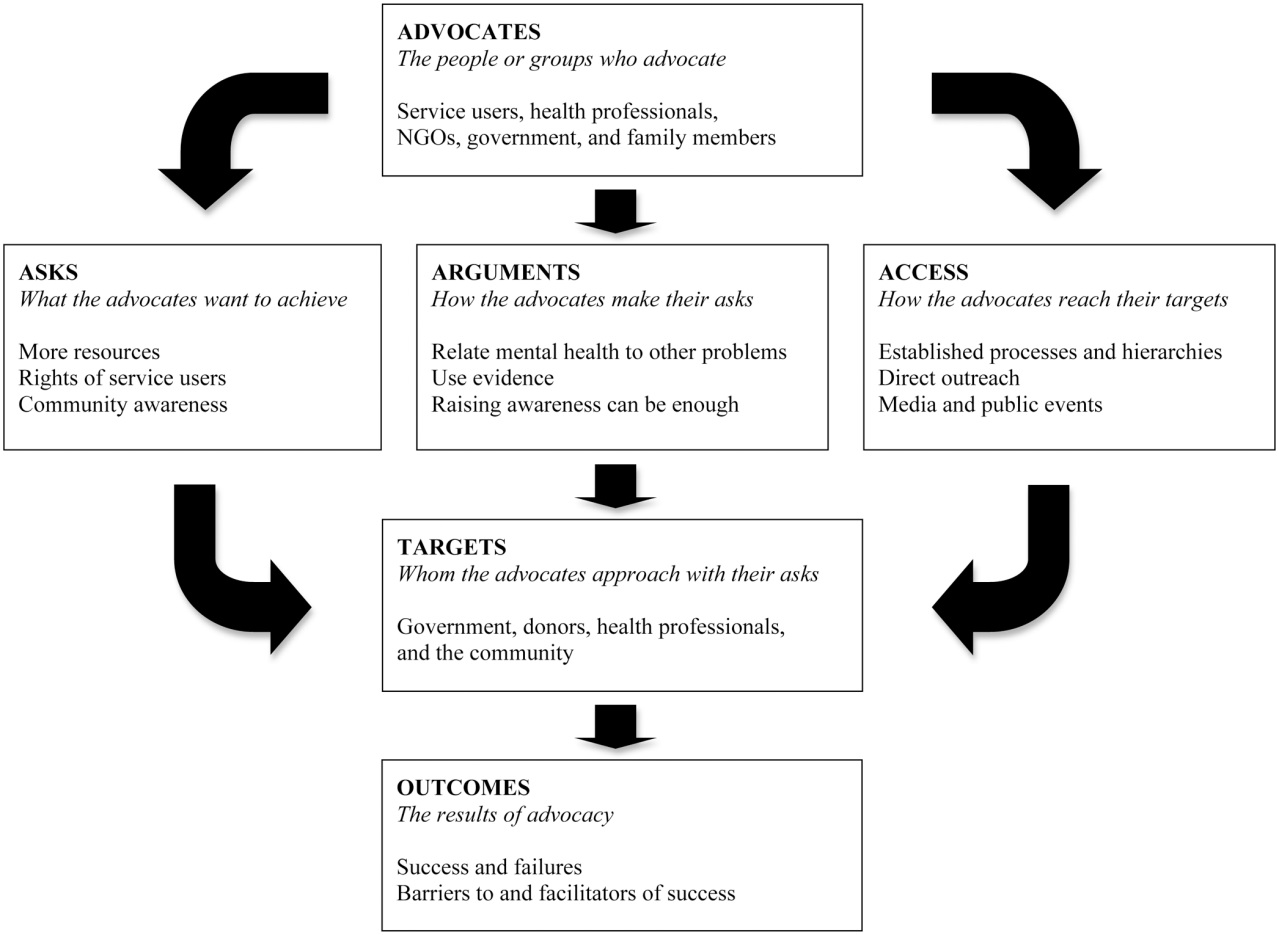
Postulated relationship between components of advocacy respondents discussed.

We categorized respondents’ comments into six areas (Table 2; organized into a flow chart in Fig 1): advocates, targets, asks, access, arguments, and outcomes. The categories that emerged from our data differ somewhat from those proposed by Farrer and colleagues in their recent meta-analysis of advocacy for health equity: evidence, who advocates and to whom, advocacy messages, tailoring arguments to different political standpoints, barriers and enables of effective advocacy, and practices that increase the effectiveness of advocacy efforts [28]. However, most ideas expressed by our respondents do fit into Farrer’s framework, which affirms that the perspectives of our participants are in line with international understandings of health advocacy. Comparison of our findings with Shiffman and Smith’s determinants of political will (advocates’ power, how the problem is understood, political contexts, and features of the problem) [12] underscores that mental health advocacy extends beyond generating political will via governmental action. While our respondents did describe advocacy to government and donors for improved policies and resource allocation, they also emphasized educating the community and healthcare providers as essential for promoting mental health.

A tension emerged from our data concerning whether respondents advocate within or outside established, formal channels. Most respondents described advocating to whoever was just above them in a hierarchy, whether a nurse to a doctor or one government official to another. In contrast, one respondent described how service users can write about their grievances directly to the president of Zimbabwe. Strategizing about where advocacy efforts should fall along this spectrum is especially important given another finding, that government is not monolithic. Diverse individuals and departments have different views about mental health and powers to influence policy and resource allocation. Respondents identified a need to build strong relationships with advocacy targets by approaching them with respect and deference. However, the more extensive the hierarchy an advocacy message must ascend to reach a decision-maker, the more opportunities for it to be rebuffed. Moreover, hierarchical decision-making processes present a differential challenge to advocates depending on their status within or position outside the mental health system.

Respondents also presented various ideas about the relationship between mental health and other areas of health services. Some respondents said that mental health care and advocacy ought to be integrated into primary care and efforts combating infectious disease, whereas others believed subsuming mental health within a larger conceptual umbrella (i.e. non-communicable diseases) led to its neglect. Respondents highlighted distinctive dimensions of mental health advocacy, including stigma, involuntary institutionalization, and politics concerning social determinants, while also drawing parallels to and inspiration from more successful Zimbabwean HIV activism [45].

Several of our findings may inform future development of mental health advocacy in Zimbabwe and other LMICs. One of the themes across interviews was the importance of involving service users in advocacy, but how rarely this happened. Our data suggest that key stakeholders might well unite around programs aimed at empowering service users to raise awareness about the importance of mental health and the need for more resources, given service users’ first hand perspectives, personal investment in effecting improvements, and compelling narratives. In designing future initiatives, policymakers, planners, and donors may find it fruitful to consider the advocacy strategies that our respondents proposed, such as gathering and presenting epidemiological data, relating mental health to more widely appreciated issues of concern, soliciting service user input on advocacy priorities, and engaging service users in advocacy efforts. Participants did not mention the need to support any service user-led advocacy efforts, but investigation of whether such efforts exist and how they can be fostered or supported is essential if service users are to be engaged as equal partners in advocacy and systems change.

At a more basic level, our findings underscore the perceived importance of including support for advocacy in initiatives aimed at strengthening mental health systems. Overall, our participants expressed that advocacy to the general public and within government is essential to overcome stigma and increase access to care. Our participants would likely be receptive to support for advocacy from governments and international donors. While they believe that advocacy is an effective change strategy, they raised few examples of specific successes, making further exploration of the effectiveness and outcomes of investment in advocacy versus direct service important prior to or alongside investment.

While this study focused on Zimbabwe, our findings may be applicable in other LMICs, since many of the challenges that Zimbabwe faces to increasing access to mental health services, including stigma, competing priorities, and lack of resources, are shared [16]. Qualitative research on mental health advocacy in other African countries has emphasized similar themes, such as service user involvement [31] and the need for better data [33], suggesting a commonality in thinking between stakeholders in Zimbabwe and other African nations. Mental health leaders in other LMIC may find the multi-dimensional framework that emerged from our data useful in evaluating the strengths and weaknesses of advocacy in their countries and identifying opportunities for high-yield interventions.

Two limitations merit particular consideration in interpreting our results. First, our interview guide asked only a few direct questions about advocacy. On the one hand, that so many participants volunteered information about advocacy increased our confidence that this topic was important to them. On the other hand, a more targeted interview might have elicited more information or different emphases. Second, we only interviewed leaders in mental health at the national level in Zimbabwe. While these perspectives are of particular importance given their authority and while we spoke with people in many areas of the country, including some rural areas, they may not represent the opinions and experience of more rank and file providers and officials, the population as a whole, or service users. Because, as our respondents emphasized, service user voices are essential, but underrepresented in advocacy, future research on this topic should actively solicit service users’ participation and leadership.

Despite the importance of health advocacy, research on the topic remains nascent. More perspectives from different voices and disciplines are needed. Future studies should evaluate different strategies and platforms for advocacy: which strategies are most effective in increasing access to mental health services and quality of care? The goal of new research should be to guide the practice of advocates as they work to improve health outcomes. Additionally, further research in LMICs is needed on ways to work with service users as equal partners, ensure that their voices are present in advocacy and development of priorities, and empower them to not only participate in, but also lead, advocacy efforts [18]. Because it runs counter to many traditional hierarchies and power structures, equitable work with service users remains a challenge throughout mental health services and should be central in development of research and advocacy priorities in LMICs [46, 47]. Specifically within Zimbabwe, we first need to better understand the perspectives of service users on advocacy and systems change.

In Zimbabwe and worldwide, most people with mental disorders do not receive the treatment they need, in large part because mental health is not prioritized. Key stakeholders in Zimbabwe’s mental health system affirm that advocacy can and should hasten awakening to this crisis. It is our hope that exploring how advocacy functions in the Zimbabwean context despite economic, political, and social barriers will contribute to the development of broader advocacy that can convince communities locally and globally of the importance of mental health.
